# G-CSFR antagonism reduces neutrophilic inflammation during pneumococcal and influenza respiratory infections without compromising clearance

**DOI:** 10.1038/s41598-019-54053-w

**Published:** 2019-11-27

**Authors:** Hao Wang, Christian Aloe, Nick Wilson, Steven Bozinovski

**Affiliations:** 1 School of Health & Biomedical Sciences, RMIT University, Bundoora, 3083 Australia; 2 CSL Limited, Parkville, Victoria, 3052 Australia

**Keywords:** Neutrophils, Infection

## Abstract

Excessive neutrophilic inflammation can contribute to the pathogenesis of pneumonia. Whilst anti-inflammatory therapies such as corticosteroids are used to treat excessive inflammation, they do not selectively target neutrophils and may compromise antimicrobial or antiviral defences. In this study, neutrophil trafficking was targeted with a granulocyte-colony stimulating factor receptor monoclonal antibody (G-CSFR mAb) during *Streptococcus pneumoniae* (serotype 19F) or influenza A virus (IAV, strain HKx31) lung infection in mice. Firstly, we demonstrated that neutrophils are indispensable for the clearance of *S. pneumoniae* from the airways using an anti-Ly6G monoclonal antibody (1A8 mAb), as the complete inhibition of neutrophil recruitment markedly compromised bacterial clearance. Secondly, we demonstrated that G-CSF transcript lung levels were significantly increased during pneumococcal infection. Prophylactic or therapeutic administration of G-CSFR mAb significantly reduced blood and airway neutrophil numbers by 30–60% without affecting bacterial clearance. Total protein levels in the bronchoalveolar lavage (BAL) fluid (marker for oedema) was also significantly reduced. G-CSF transcript levels were also increased during IAV lung infection. G-CSFR mAb treatment significantly reduced neutrophil trafficking into BAL compartment by 60% and reduced blood neutrophil numbers to control levels in IAV-infected mice. Peak lung viral levels at day 3 were not altered by G-CSFR therapy, however there was a significant reduction in the detection of IAV in the lungs at the day 7 post-infection phase. In summary, G-CSFR signalling contributes to neutrophil trafficking in response to two common respiratory pathogens. Blocking G-CSFR reduced neutrophil trafficking and oedema without compromising clearance of two pathogens that can cause pneumonia.

## Introduction

Pneumonia remains a major global health problem, resulting in high mortality rates in children younger than 5 years of age, the elderly and people with lung diseases such as chronic obstructive pulmonary disease (COPD) and asthma^1–3^. The major pathogens that cause pneumonia include *Streptococcus pneumoniae* (SP) and influenza A virus (IAV), which account for over 30% and 10% of pneumonia deaths, respectively^4^. Despite the broad use of antibiotics and vaccines, the incidence of lung infections caused by SP and IAV remain high in susceptible populations including patients with chronic lung conditions and represents a major burden to healthcare systems^5^. The early innate immune response to lung infection plays an essential role in clearance of pathogens involving rapid mobilisation of neutrophils into the lungs. However, pathogenic strains can also initiate excessive neutrophil trafficking into the lungs that causes collateral lung damage and remodelling. One of the major therapeutic challenges in targeting excessive inflammation is that the treatment may compromise clearance of respiratory pathogens. For example, the use of inhaled corticosteroids is associated with increased risk of pneumonia hospitalization among elderly patients with COPD^6^.

Since corticosteroids elicit multiple anti-inflammatory and immunosuppressive actions, more selective targeting of distinct immune cell populations may reduce the burden of lower respiratory tract infections (LRIs). Neutrophils dominate the early innate immune response during bacterial and viral infections. The influx of neutrophils into the airways is normally self-limiting, as short-lived neutrophils undergo apoptosis and are subsequently phagocytised by alveolar and exudative macrophages in the lungs. However, despite their critical role in pathogen containment, excessive neutrophil activation will generate reactive oxygen species (ROS) and release a variety of proteases that can degrade extracellular matrix, resulting in acute lung injury and pulmonary oedema. Additionally, neutrophil extracellular traps (NETs) that are released into extracellular space to capture and kill pathogens during infection may further contribute to pathological lung damage when in excess^7,8^.

The current literature suggests that depletion of neutrophils during acute respiratory infections will leave the host susceptible to acute influenza infection. The broad depletion of circulating and tissue neutrophils with a monoclonal antibody (1A8 clone) that binds to Ly6G caused neutropenia in mice infected with influenza A virus, which subsequently developed more severe disease and higher rates of mortality^9^. However, there is conflicting data in the pneumococcal lung infection models, where serotype appears to dictate whether neutrophils are indispensable for bacterial clearance. Depletion of neutrophils has been shown to be beneficial during infection with the invasive serotype 8 as the degree of pneumonia and septicaemia was reduced, which resulted in prolonged survival^10^. In contrast, neutrophil depletion resulted in markedly higher bacterial loads in the lungs and blood of mice infected with the invasive serotype 2 pneumococci^11^. It is not known whether clearance of the less invasive and more common 19F pneumococcal serotype will be affected by neutrophil depletion, which will be addressed in our study.

Since the depletion of circulating and tissue neutrophils may compromise respiratory pathogen clearance, targeting excessive neutrophil trafficking may provide a safer therapeutic approach. Chemoattractants including interleukin (IL)-8/C-X-C motif ligand 8 (CXCL8), CXCL1, CXCL2 and CXCL5 released from alveolar epithelial cells and macrophages^12^ promote neutrophil transmigration into the air spaces to clear invading pathogens. In addition, the hematopoietic growth factor granulocyte colony-stimulating factor (G-CSF) contributes to neutrophil granulopoiesis in the bone marrow and promotes neutrophil trafficking by modulating chemokine and adhesion receptors (CXCR2 and CD62L) on neutrophils^13^. Neutralising G-CSF in a mouse pneumococcal model has been shown to reduce lung neutrophil numbers without causing outgrowth of invasive pneumococci (serotype 3) in the lungs^14^. However, anti-G-CSF reduced blood neutrophil counts by over 50% in uninfected mice^14^, which may leave the host susceptible to secondary infections. An alternative approach is the inhibition of its receptor, G-CSF receptor (G-CSFR). An advantage of this approach is that neutralizing G-CSFR does not reduce circulating neutrophil numbers in healthy nonhuman primates with repeated doses and over prolonged periods^15^. In addition, G-CSFR mAb therapy during influenza infection in mice did not cause neutropenia or compromise viral clearance, although it was not established whether viral-induced blood neutrophilia and neutrophil trafficking into the lungs were reduced. In our study, we have investigated how alternative neutrophil blocking strategies (1A8 vs. anti-G-CSFR monoclonal antibody) impact on lung injury/oedema and clearance of serotype (19F) pneumococcus, which readily colonizes the airways^16^. We have also assessed whether G-CSFR mAb can safely reduce neutrophilic lung inflammation and injury/oedema during acute IAV infection in mice.

## Results

### Neutrophil depletion with 1A8 mAb markedly worsens pneumococcal infection outcomes

**Figure 1:**
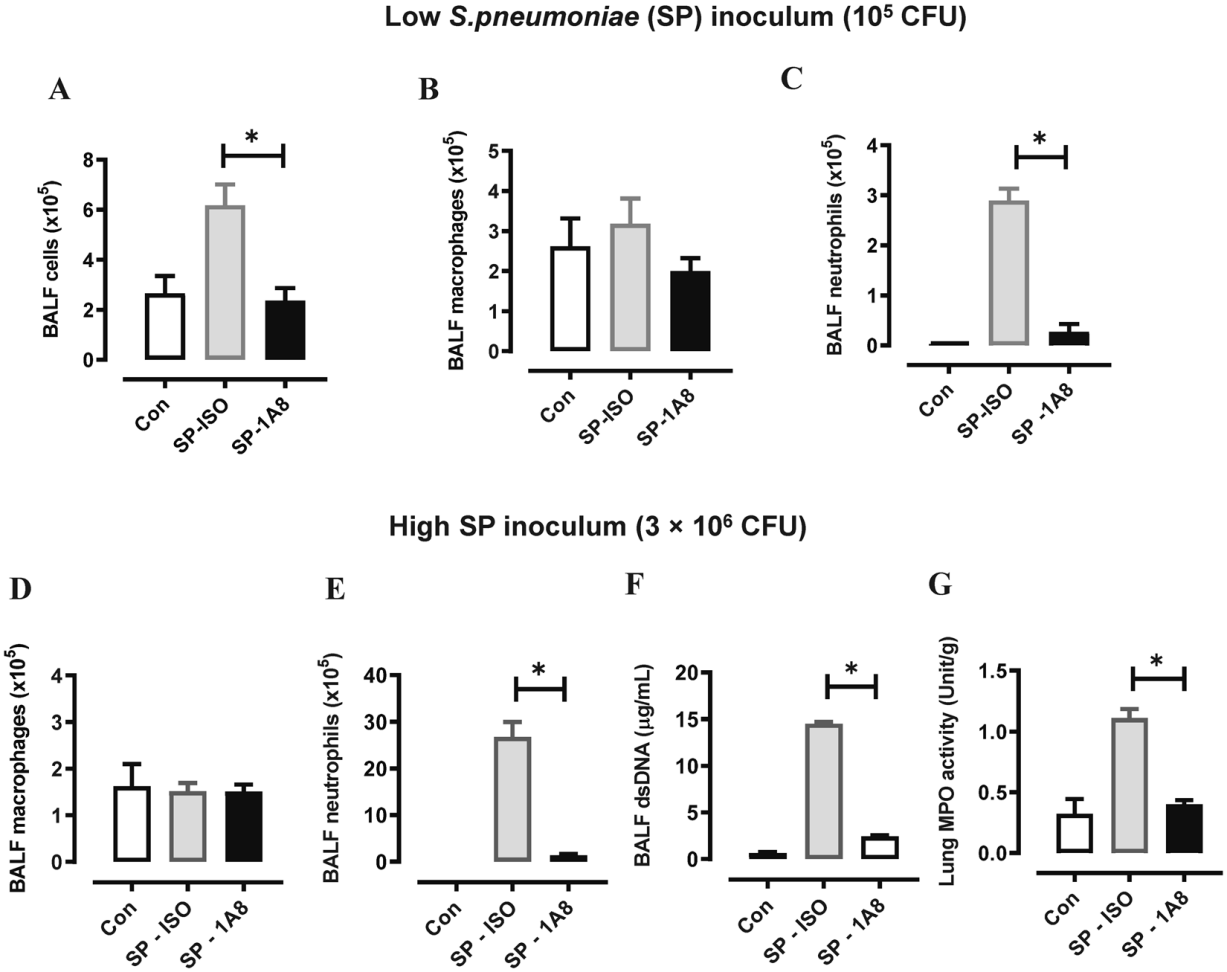
1A8 mAb potently suppressed BAL neutrophil numbers during SP infection. (**A**–**C**) Mice were inoculated with a low inoculum of SP (10^5^ CFU) and treated with 1A8 mAb or isotype control (ISO) one day prior and one day after SP infection. At day 2 post SP infection, bronchoalveolar lavage (BAL) was performed. (**A**) The total BAL cell count was increased in SP-infected mice and 1A8 treatment significantly reduced this response to control/uninfected levels. (**B**) Differential cell counts were performed and showed that BAL macrophages were not significantly altered by SP infection or 1A8 treatment. (**C**) BAL neutrophil numbers were markedly increased in response to SP infection and 1A8 mAb treatment significantly suppressed this response by over 90%. (**D**–**F**) Mice were inoculated with a high inoculum of SP (3 × 10^6^ CFU) and treated with 1A8 mAb or isotype control (ISO) one day prior and one day after SP infection. At day 1 post SP infection BAL was performed, and differential counts showed that (**D**) BAL macrophages were not significantly altered by SP infection or 1A8 treatment. (**E**) BAL neutrophil numbers were markedly increased in response to SP infection and 1A8 mAb treatment significantly suppressed this response by over 90%. (**F**) Assessment of net dsDNA levels in the BALF was used a marker for netosis, where levels were markedly increased by SP-ISO infection, and 1A8 mAb treatment significantly reduced dsDNA levels by over 90%. (**G**) Lung myeloperoxidase (MPO) activity was quantified as a marker for tissue neutrophil content, where increased activity in SP-ISO treated mice was significantly reduced by 1A8 mAb to control/uninfected levels. n = 5–6 ^*^p < 0.05, one-way ANOVA.

**Figure 2:**
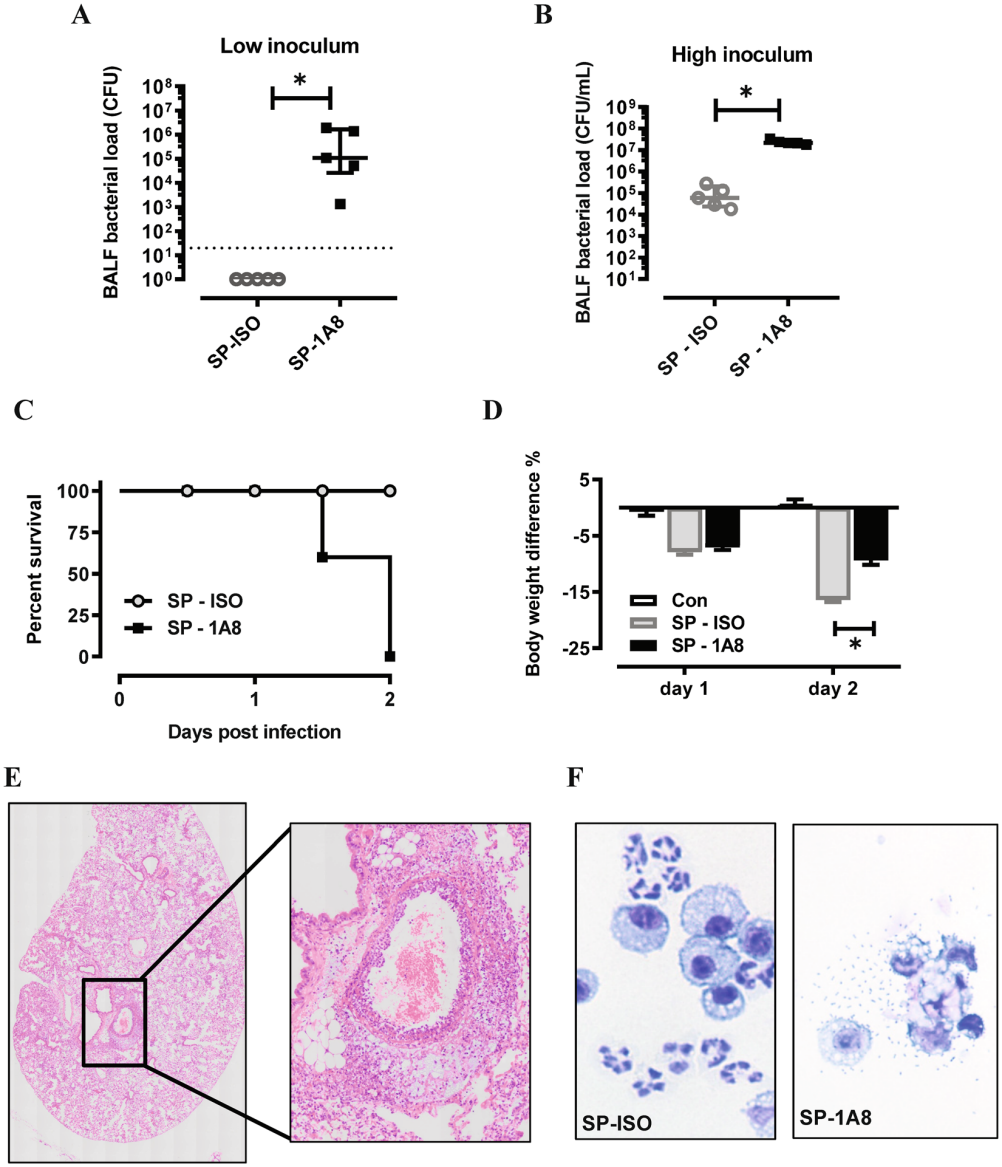
Neutrophil depletion by 1A8 mAb resulted in uncontrolled bacterial growth in the lungs. (**A**,**B**) Lung bacterial load was quantified by serially diluting BALF on horse blood agar supplemented with 5 µg/ml gentamicin. Following overnight incubation at 37 °C, colony forming units (CFUs) were recorded from the highest-countable dilution. (**A**) Pneumococci in the BAL was cleared by day 2 in the low inoculum model (10^5^ CFU), whereas 1A8 mAb resulted in markedly higher BAL bacterial counts. Dotted line denotes detection limit. (**B**) In the high inoculum model, SP was detected in the BALF of isotype antibody (ISO) treated mice at day 1 post infection and levels were increased over 2-log in the 1A8 mAb treated mice. n = 5–6, *p < 0.05, two-tailed Students t-tests. (**C**) Kaplan-Meier survival curve analysis demonstrated that all mice inoculated with high dose SP and treated with isotype antibody treated (SP-ISO) mice survived, whereas 1A8 mAb antibody treatment (SP-1A8) mice resulted in 100% mortality by day 2. (**D**) High inoculum SP infection resulted in body weight loss on day 1 and day 2 in ISO treated mice, and the degree of loss was significantly reduced in 1A8 mAb treated mice. n = 5–6, *p < 0.05, one-way ANOVA. (**E**) Representative whole slide scan H&E image of lung lobe from SP-1A8 treated mouse on day 2 post high inoculation with insert, identifying significant perivascular and pulmonary oedema with cocci present in this region. (**F**) Representative image of BAL cells from SP-1A8 mice on day 2 post inoculation identified necrotic macrophages surrounded by pneumococci, which was absent from SP-ISO treated mice.

To determine whether neutrophils are indispensable for the clearance of *S. pneumoniae* 19F serotype in the lung, we adopted an established method^11^ to deplete neutrophils using a 1A8 mAb (anti-Ly6G). The efficacy of 1A8-mediated neutrophil depletion was firstly evaluated in a low inoculum model whereby mice were infected with 10^5^ CFU *S. pneumoniae* (Fig. 1A–C) or high inoculum at 3 × 10^6^ CFU *S. pneumoniae* (Fig. 1D–F). Evaluation of BALF cellularity in the low inoculum model demonstrated that total cell numbers were reduced with 1A8 mAb treatment at day 2 post infection (Fig. 1A), where 1A8 mAb did not alter macrophage numbers (Fig. 1B), but markedly reduced neutrophil numbers by over 90% (Fig. 1C). In the high inoculum model, BALF macrophage numbers (Fig. 1D) were not altered at day 1 post infection, but there was a significant and potent reduction in BALF neutrophils (Fig. 1E). We also measured dsDNA in BALF as a surrogate marker for netosis and demonstrate that dsDNA levels markedly increased in response to high inoculum SP infection (Fig. 1F). Furthermore, dsDNA levels in SP-infected mice were almost reduced to control levels by neutrophil depletion, which validates neutrophils as the predominant source of extracellular dsDNA in this model. In addition, lung MPO activity was measured as a surrogate for lung neutrophil content, where levels were increased by SP-infection and reduced to control levels by 1A8-mediated neutrophil depletion (Fig. 1G). Having demonstrated that 1A8 potently suppressed lung neutrophil trafficking, we next established how this affected bacterial clearance and lung pathology. In the low inoculum model, *S. pneumoniae* was effectively cleared in the lungs, however suppression of neutrophil trafficking with 1A8 mAb resulted in a high bacterial burden in the lungs at day 2 post *S. pneumoniae* infection (Fig. 2A). A similar result was seen in the high inoculum model, whereby 1A8 mAb treatment resulted in a greater than 2-log increase in pneumococcal load at day 1 post *S. pneumoniae* infection (Fig. 2B).

Consequent to increased bacterial burden in the high *S. pneumoniae* (SP) inoculum model, 1A8 mAb treated mice displayed more severe clinical symptoms with abnormal gait and laboured breathing leading to 100% mortality by day 2, in contrast to isotype-treated (SP-ISO) mice that all survived (Fig. 2C). Mortality was not caused by excessive weight loss, rather 1A8 mAb treated mice displayed significantly less body weight loss at day 2 post infection (Fig. 2C). An independent pathology report of the mice that reached ethical endpoint concluded that there was marked perivascular oedema with bacterial cocci present. A representative image of the lung pathology (Fig. 2E), identified prominent perivascular and pulmonary oedema. Multifocally, there are areas of necrosis of the parenchyma and at the centre of some of these areas, vasculitis and microthrombosis of the capillary was observed. A representative cytospin image of the BAL cells identified morphological changes in macrophages from SP-1A8 treated mice associated with ruptured membrane typical of pyroptotic or necroptotic cells, which are likely to be driven by the high bacterial levels and toxaemia (Fig. 2F). The pathology reports also reported evidence for bacterial dissemination to the heart involving multifocal fibrinous epicarditis with bacterial cocci present, and the spleen was markedly congested with noticeable lymphocytolysis, suggestive of toxaemia.

### G-CSFR mAb reduces neutrophilic inflammation without compromising bacterial clearance

**Figure 3:**
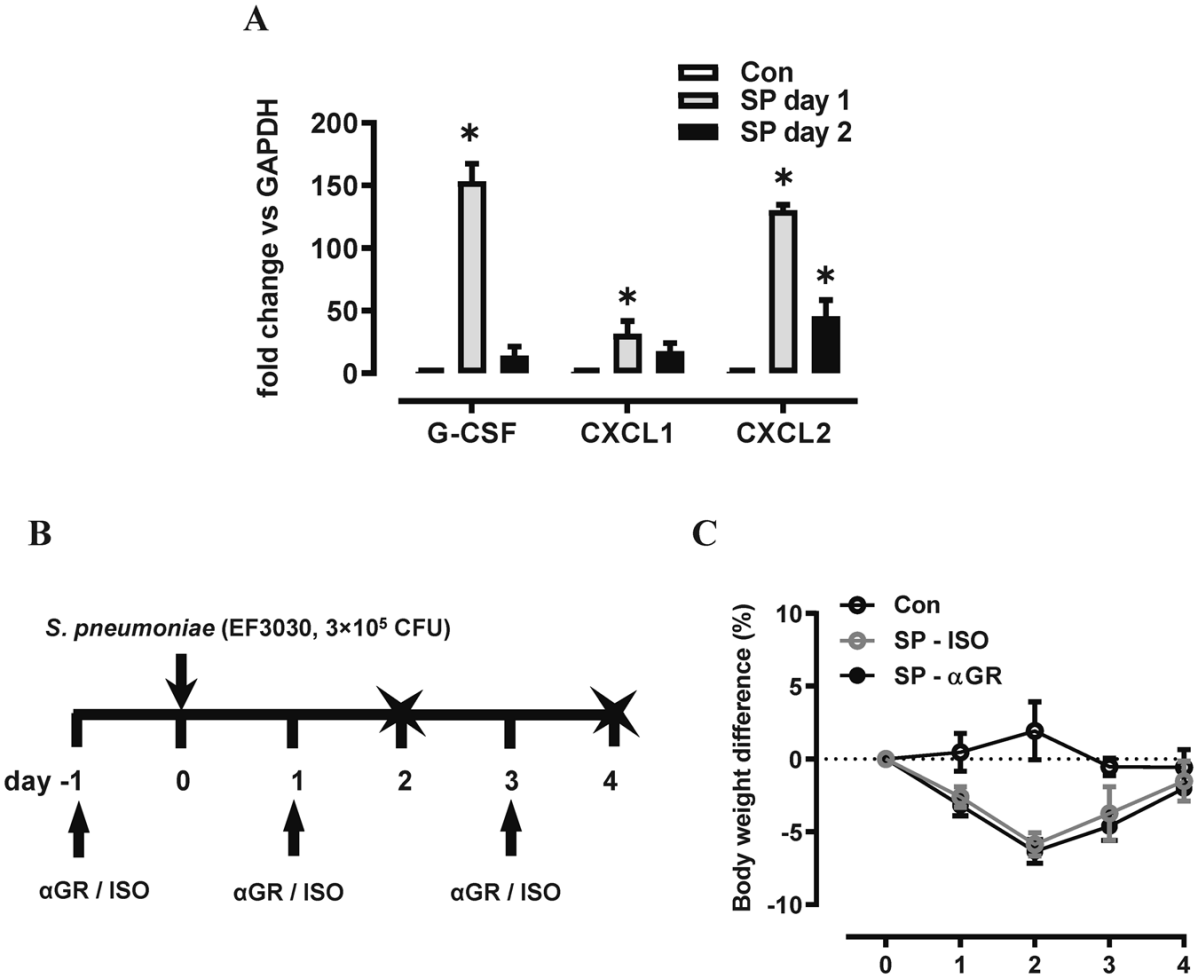
G-CSF expression is increased in the lungs of mice infected with *S. pneumoniae* (SP). (**A**) RTqPCR analysis of neutrophil chemokines in lung tissue identified a significant increase in CXCL1, CXCL2 and G-CSF transcript levels at day 1 post high inoculum (3 × 10^6^ CFU) SP infection. n = 4–5, *p < 0.05 vs Con, one-way ANOVA. (**B**) Schematic diagram of the αG-CSFR experimental protocol, where mice were infected with SP using a moderate inoculation dose (3 × 10^5^ CFU) and treated with anti-G-CSFR (αGR) or isotype antibody at the indicated timepoints. Endpoint measurements were performed at day 2 and day 4 post SP infection (denoted with X) . (**C**) SP-ISO infection resulted in 5% body weight loss by day 2 with recovery by day 4, and this response was not altered by αGR (n = 5–10 per group).

**Figure 4:**
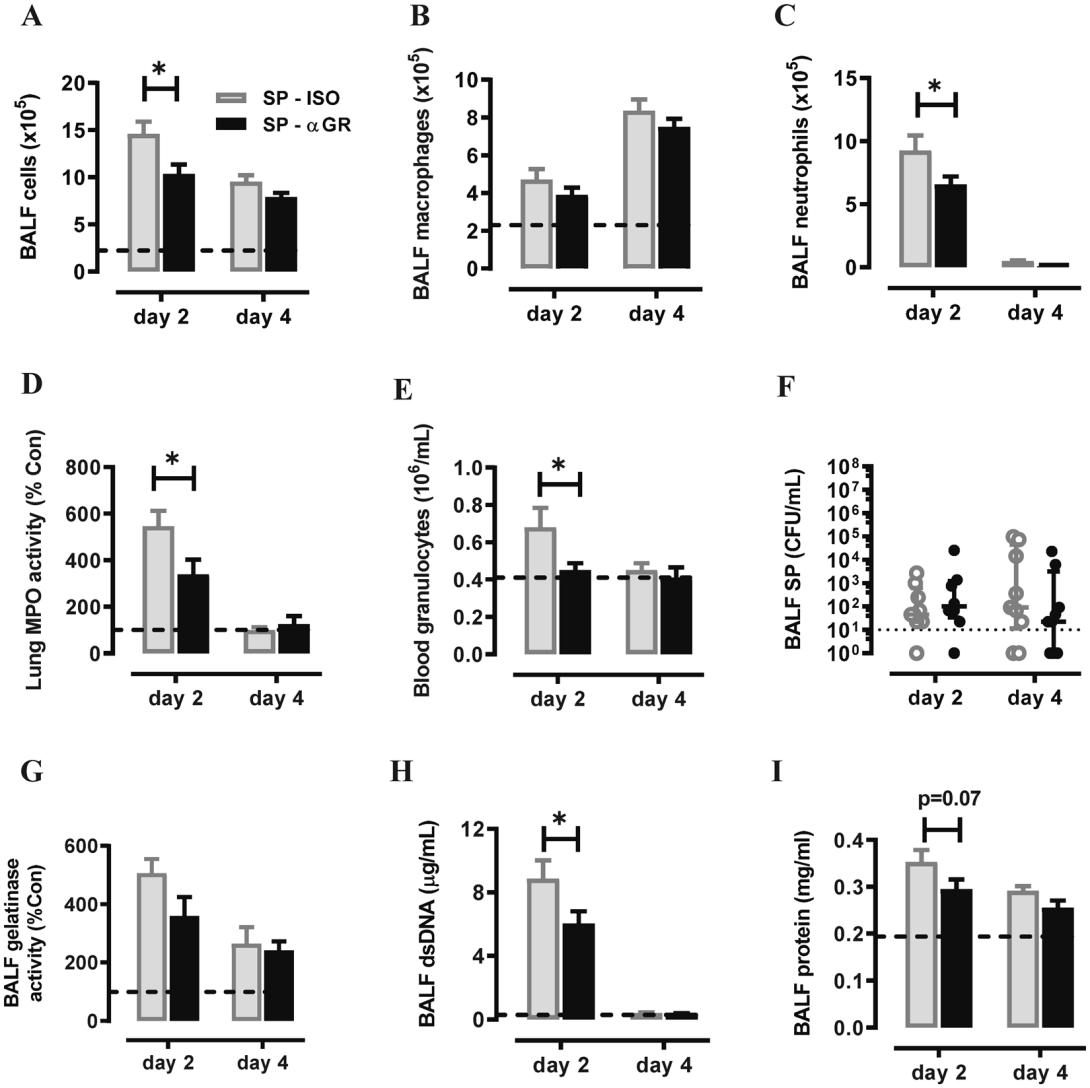
Prophylactic administration of αGR mAb reduced neutrophil numbers without worsening pneumococcal lung load. (**A**–**C**) Total and differential BAL cell counts were performed on mice infected with a moderate dose of SP and prophylactically treated αGR, as detailed in (**B**). The dotted line in each graph represents control/uninfected levels for each parameter assessed (**A**) The total BAL cell count was increased in SP-ISO infected mice and αGR mAb treatment significantly reduced this response at day 2 but not day 4. (**B**) Differential cells counts were performed and showed that BAL macrophages were elevated at day 4 post SP infection, but αGR mAb treatment did not alter this response. (**C**) BAL neutrophil numbers were markedly increased in response to SP infection at day 2 and αGR mAb treatment significantly suppressed this response by 30%. (**D**) Myeloperoxidase (MPO) activity in lung tissue was also increased in SP-ISO treated mice at the peak day 2 timepoint and αGR mAb treatment significantly reduced this response by approximately 40%. (**E**) Blood was collected via cardiac puncture and granulocytes were quantified on a Cell-Dyn Emerald Hematology Analyzer, which showed that an elevated granulocyte count at day 2 was reduced to control/uninfected levels with αGR mAb treatment. (**F**) Bacterial load in the BAL compartment was quantified on HBA plates and demonstrated that treatment with αGR mAb did not alter pneumococcal lung growth or clearance relative to ISO treated mice. (**G**) Assessment of net gelatinase activity in the BALF identified day 2 as the peak of activity, and αGR mAb treatment did not significantly alter this activity. (**H**) Assessment of net dsDNA levels in the BALF was used a marker for netosis, where levels peaked at day 2 post SP infection, and αGR mAb treatment significantly reduced peak dsDNA levels. (**I**) Assessment of total protein levels in the BALF was used a marker for oedema, where levels peaked at day 2 post SP infection, and αGR mAb treatment did not significantly alter peak levels. n = 5–10, *p < 0.05, two-way ANOVA.

We subsequently measured G-CSF, CXCL1 and CXCL2 transcript levels in the lungs during high inoculum pneumococcal infection. All three neutrophil trafficking mediators were significantly increased on day 1 post infection, with G-CSF being the highest (154-fold, Fig. 3A). We next examined the efficacy and safety of neutralizing G-CSFR during pneumococcal infection, firstly using a prophylactic approach in mice infected with a moderate dose of *S. pneumoniae* (3 × 10^5^ CFU). Mice were i.p. injected with anti-G-CSFR mAb (αGR) or isotype control antibody (ISO) on day -1, 1, and 3 post SP infection. Outcomes were assessed at the acute infection phase (day 2) and recovery phase (day 4) as summarised in Fig. 3B. αG-CSFR treatment did not alter body weight loss caused by pneumococcal infection (Fig. 3C). However, there was a 30% reduction in both BALF total cells on day 2 (Fig. 4A), where BALF macrophage numbers were not altered (Fig. 4B), but there was a significant 30% reduction in BAL neutrophil numbers (p < 0.05, Fig. 4C). At the later day 4 recovery phase, neutrophils were no longer recovered in the BALF and there was a 2-fold increase in BALF macrophages in pneumococci-infected mice, which was not altered by αG-CSFR treatment (Fig. 4B,C). αG-CSFR treatment also effectively reduced lung neutrophilic inflammation, demonstrated by a significant reduction in myeloperoxidase (MPO) activity (Fig. 4D). Blood neutrophil numbers were also quantified, demonstrating an increase in SP-infected mice at day 2, and αG-CSFR treatment normalised the elevated blood granulocyte count (Fig. 4E). In addition, no difference in BALF pneumococcal load was observed on both day 2 and day 4 in αG-CSFR-treated mice relative to the isotype-treated control mice (Fig. 4F). We next measured markers of neutrophil activation (gelatinase activity), netosis (dsDNA) and pulmonary oedema (total protein levels) in the BALF. This data shows that *S. pneumoniae* infection increased gelatinase activity (Fig. 4G), dsDNA levels (Fig. 4H) and total protein levels (Fig. 4I) in the BALF relative to control/uninfected mice (dotted line). Of note, αG-CSFR-treatment significantly reduced dsDNA levels and there was a trend towards reduced gelatinase activity and protein levels at the peak day 2 timepoint.

**Figure 5:**
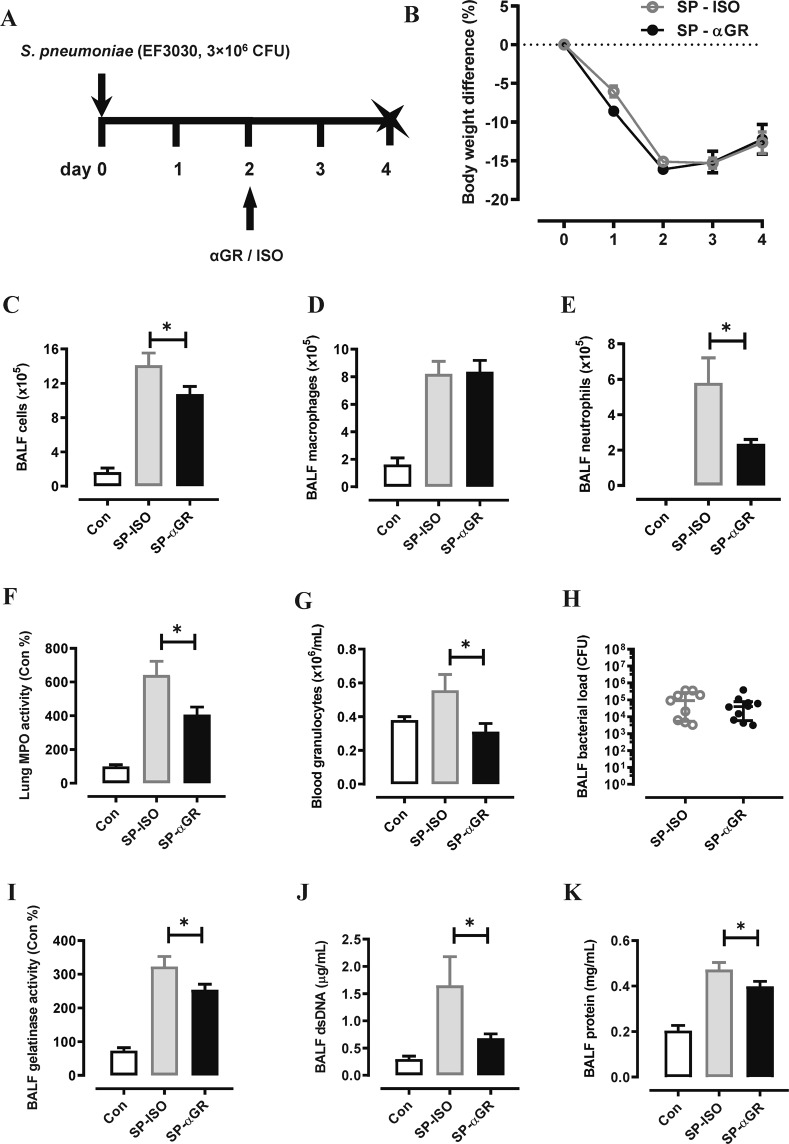
Therapeutic administration of αGR mAb reduced neutrophil numbers and oedema without worsening pneumococcal lung load. **(A)** Schematic diagram of the αG-CSFR experimental protocol, where mice were infected with SP using a high inoculation dose (3 × 10^6^ CFU) and treated with anti-G-CSFR (αGR) or isotype antibody at the indicated timepoint. Endpoint measurements were performed at day 4 post SP infection (denoted with X) . (**B**) SP-ISO infection resulted in >15% body weight loss by day 2 with partial recovery by day 4, and this response was not altered by αGR treatment (n = 8–10 per group). (**C**–**E**) Total and differential BAL cell counts were performed on mice infected with a high dose of SP and therapeutically treated with αGR, as detailed in (**A**). (**C**) The total BAL cell count was increased in SP-ISO infected mice and αGR mAb treatment significantly reduced this response at day 4. (**D**) Differential cell counts were performed and showed that BAL macrophages were elevated at day 4 post SP infection, but αGR mAb treatment did not alter this response. (**E**) BAL neutrophil numbers were increased in response to SP infection at day 4 and αGR mAb treatment significantly suppressed this response by 60%. (**F**) Myeloperoxidase (MPO) activity in lung tissue was also increased in SP-ISO treated mice and αGR mAb treatment significantly reduced this response by approximately 40%. (**G**) There was an elevated granulocyte count in SP-ISO infected mice and this was reduced to control/uninfected levels with αGR mAb treatment. (**H**) Bacterial load in the BAL compartment was quantified and demonstrated that αGR mAb treatment did not alter pneumococcal lung growth or clearance relative to ISO treated mice. (**I**) Assessment of net gelatinase activity in the BALF showed elevated activity by SP infection and αGR mAb treatment significantly reduced this activity. (**J**) The netosis marker (dsDNA) was elevated by SP infection in the BALF and αGR mAb treatment significantly reduced this response. (**K**) The oedema marker (total protein levels in the BALF) was increased by SP infection and αGR mAb treatment significantly reduced this response. n = 5–10, *p < 0.05, one-way ANOVA.

Having established that αG-CSFR mAb is safe and effective during pneumococcal infection when administrated prophylactically, we next assessed αG-CSFR in a therapeutic setting. In this model, mice were infected with a high inoculum of pneumococcus (3 × 10^6^ CFU) and αG-CSFR or isotype mAb was administered at the peak day 2 post infection timepoint, and outcomes were assessed at the later day 4 recovery phase (Fig. 5A). Consistent with the prophylactical model, αG-CSFR therapeutic treatment did not alter body weight loss (Fig. 5B) but did significantly reduce total BALF cells (Fig. 5C) and BALF neutrophils (Fig. 5E), without altering macrophage numbers (Fig. 5D). αG-CSFR treatment also markedly reduced MPO/neutrophilic inflammation in the lung (Fig. 5F) and in the circulation (Fig. 5G), without compromising pneumococcal clearance (Fig. 5H). In addition, markers of neutrophil activation (gelatinase activity, Fig. 5I), netosis (dsDNA, Fig. 5J) and pulmonary oedema (total protein levels, Fig. 5K) in the BALF were all significantly reduced by αG-CSFR administration.

### Neutralizing G-CSFR reduces neutrophilic inflammation without compromising viral clearance

**Figure 6:**
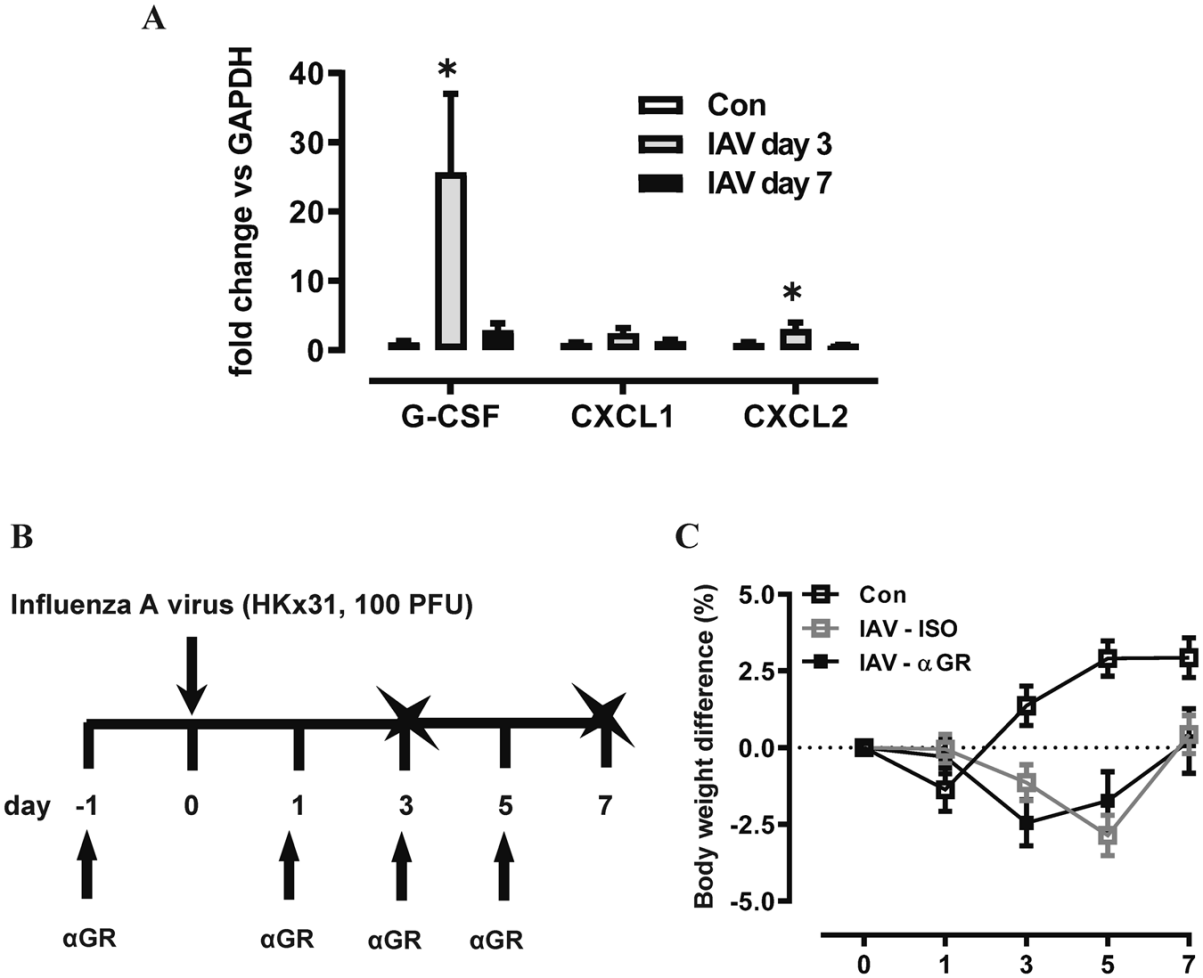
G-CSF expression is increased in the lungs of mice infected with Influenza A virus (IAV). (**A**) RTqPCR analysis of neutrophil chemokines in lung tissue identified a significant increase in G-CSF and CXCL2 transcript levels at day 3 post IAV infection. n = 5–8, *p < 0.05 vs Con, one-way ANOVA. (**B**) Schematic diagram of the αG-CSFR experimental protocol, where mice were infected with IAV (100 PFU) and treated with anti-G-CSFR (αGR) or isotype antibody at the indicated timepoints. Endpoint measurements were performed at day 3 and day 7 post IAV infection (denoted with X). (**C**) IAV-ISO infection resulted in modest body weight loss by day 3 with recovery by day 7, and this response was not altered by αGR treatment (n = 5–10 per group).

**Figure 7:**
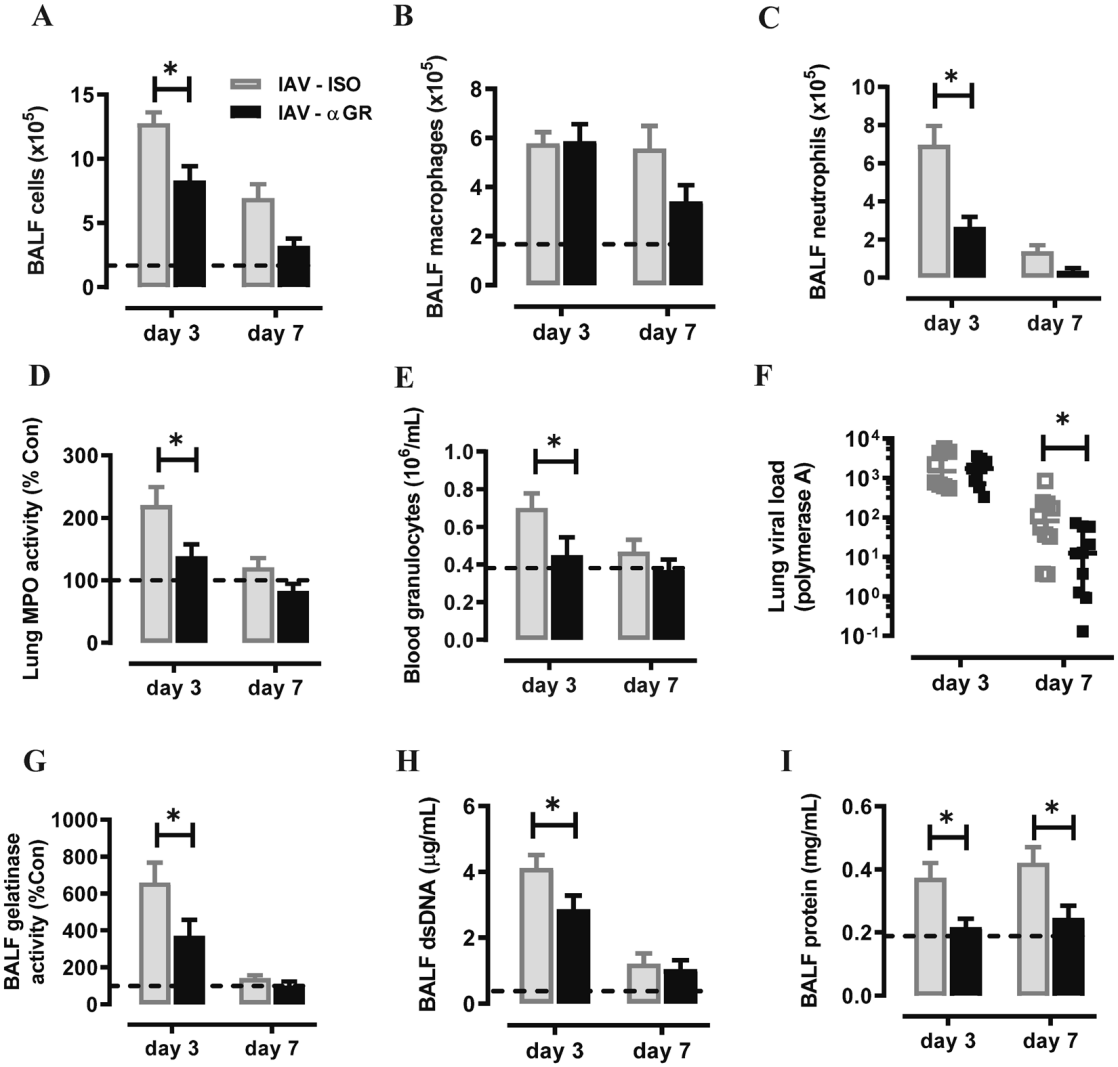
αGR mAb reduced neutrophil numbers without compromising viral clearance. (**A**–**C**) Total and differential BAL cell counts were performed on mice infected with IAV and treated αGR, as detailed in Fig. 6B. The dotted line in each graph represents control/uninfected levels for each parameter assessed (**A**) The total BAL cell count was increased in IAV-ISO infected mice and αGR mAb treatment significantly reduced this response at day 2 and day 4. (**B**) Differential cells counts were performed and showed that BAL macrophages were peaked at day 3 post IAV infection, but αGR mAb treatment did not significantly alter this response. (**C**) BAL neutrophil numbers were markedly increased in response to IAV infection at day 3 and αGR mAb treatment significantly suppressed this response by 60%. (**D**) Myeloperoxidase (MPO) activity in lung tissue was also increased in IAV-ISO treated mice at the peak day 3 timepoint and αGR mAb treatment significantly reduced this response by approximately 40%. (**E**) Blood was collected via cardiac puncture and an elevated granulocyte count at day 3 was reduced to control/uninfected levels with αGR mAb treatment. (**F**) Viral lung levels determined by RTqPCR demonstrated that treatment with αGR mAb did not alter viral at the peak day 3 timepoint, however did significantly reduce levels at the day 7 resolution timepoint (**G**) Assessment of net gelatinase activity in the BALF identified day 3 as the peak of activity, and αGR mAb treatment significantly reduced this activity. (**H**) Assessment of net dsDNA levels in the BALF identified day 3 post IAV infection as the peak, and αGR mAb treatment significantly reduced peak dsDNA levels. (**I**) Assessment of total protein levels in the BALF was used a marker for oedema, where elevated protein levels at day 3 and 7 post IAV infection were significantly reduced to control/uninfected levels with αGR mAb treatment. n = 6–10, *p < 0.05, one-way ANOVA.

We next assessed αG-CSFR therapy in an IAV lung infection model, where mice were inoculated with 100 PFU HKx31 and treated with αG-CSFR or isotype antibody. Firstly, gene expression analysis revealed significantly increased G-CSF (26-fold) and CXCL2 (3-fold) transcript levels in the lungs on day 3 post IAV infection (both p < 0.05 vs Con), with no significant change in CXCL1 levels (Fig. 6A). Outcomes were assessed during the acute (day 3) and recovery phase of infection (day 7) as summarised in Fig. 6B. No difference in body weight was observed throughout the experimental protocol between αG-CSFR-treated or isotype-treated mice (Fig. 6C). IAV infection resulted in high number of immune cells recruited in the BAL compartment, peaking on day 3 (12.8 × 10^5^/mL), and numbers reduced by 40% on day 7 (6.9 × 10^5^/mL) in isotype-treated mice (Fig. 7A). αG-CSFR treatment reduced total BALF cell numbers at both time points, by 35% on day 3 and 55% on day 7 (Fig. 7A, p < 0.05 vs IAV-ISO). BALF macrophages were not significantly altered (Fig. 7B), but there was a marked reduction in neutrophil numbers in αG-CSFR treated mice, reaching 62% on day 3 and 74% on day 7 (Fig. 7C, p < 0.05 vs IAV-ISO on day 3). Neutralizing G-CSFR also significantly reduced lung MPO activity and reduced elevated blood granulocytes to control levels (p < 0.05, IAV-αG-CSFR vs IAV-ISO, Fig. 7D,E). It was previously reported that anti-G-CSFR treatment did not alter viral titres in the lung 7 days post influenza A infection in C57BL/6 mice^17^. In our Balb/c mouse model, we did not detect any difference in the viral PA gene expression in αG-CSFR treated mice on day 3, however there was a significant reduction in viral PA gene expression on day 7, suggesting more efficient viral clearance (Fig. 7F). We next evaluated how αG-CSFR therapy affected neutrophil activation and oedema markers in the BALF. This data demonstrates that αG-CSFR therapy significantly suppressed gelatinase activity (Fig. 7G), dsDNA levels (Fig. 7H) and total protein levels (Fig. 7I) that were elevated in IAV-treated mice at the peak day 3 timepoint.

## Discussion

In this study, we have shown that G-CSFR signalling contributes to neutrophil granulopoiesis and trafficking into the airways in response to two common respiratory pathogens, namely *Streptococcus pneumoniae* and influenza A virus. Antagonising G-CSFR signalling reduced elevated blood neutrophil numbers during acute respiratory infections without causing neutropenia. In addition, neutrophil numbers in the airways were reduced in response to G-CSFR antagonism and this importantly, reduced oedema without compromising pathogen clearance. Our data shows that G-CSFR signalling contributes to neutrophil trafficking in both bacterial and viral infection models. Suppressing G-CSFR signalling can also reduce neutrophilic inflammation and rapidly halt the progression of established disease in a chronic model of inflammatory arthritis^17^. In addition, we have shown that blocking G-CSFR signalling was very effective in reducing neutrophilic inflammation in an early life infection-initiated asthma model. By selectively blocking G-CSFR, we were able to reveal that pathological remodelling of the airways underlying elevated airways hyper-responsiveness was dependent on G-CSF/G-CSFR-mediated neutrophil trafficking^18^. Our current study demonstrates that the suppression of G-CSFR signalling does not compromise host immunity to two major respiratory pathogens, in contrast to broader anti-inflammatory agents such as corticosteroids that are routinely used to treat patients with chronic inflammatory conditions such as asthma and arthritis. Hence, G-CSFR may be a safer and more targeted approach towards reducing lung injury in chronic inflammatory conditions where neutrophils dominate because it does not broadly suppress host innate immunity.

We also demonstrate that 1A8-mediated depletion of circulating and tissue neutrophils results in more severe pneumonia in response to 19F serotype infection, which is widely recognised as an upper airway colonizer that does not readily invade the bloodstream^19,20^. Our data demonstrated that broad depletion of neutrophils resulted in loss of pneumococcal clearance, which proved to be lethal at higher infection doses. The severe lung damage and mortality induced by neutrophil depletion and uncontrolled bacterial growth was likely a result of uncontrolled release of pathogenic products from *S. pneumoniae*, including hydrogen peroxide, puenumolysin and autolysin^21–23^. The dependence of neutrophils may also extend beyond their phagocytic and bactericidal function, as neutrophils actively regulate immunity during acute infection. For example, neutrophils crosstalk with macrophages by releasing neutrophil-derived TNF-related apoptosis-inducing ligand (TRAIL), which binds receptors expressed on alveolar macrophages to induce apoptosis, an important mechanism to promote pneumococcal clearance and limit inflammation^24,25^. Recruited neutrophils also produce CCL17 during infection that regulates invariant natural killer T (iNKT) cell extravasation from the vasculature, and iNKT cells have a pivotal protective role against infectious agents, particularly *S. pneumoniae*^26^.

The role of neutrophils during pneumococcal infection highlights a fine balance, where neutrophils are needed to facilitate bacterial clearance, but this response needs to be controlled in order to limit excessive neutrophil trafficking that can damage the infected tissue. Antagonists that block chemokines such as CXCL1 have been shown to reduce pathological inflammation and improve bacterial clearance in response to infection with *Pseudomonas aeruginosa*^27^. In contrast, antagonists that block the related chemokine receptor CXCR2 during pneumococcal infection suppressed both neutrophils and exudative macrophages, resulting in a failure to control pneumococcal load^28^. Likewise, suppression of interleukin-17 (IL-17), which is a key cytokine that regulates inflammation, resulted in reduced neutrophil trafficking but enhanced mortality in response to serotypes 3 and 6B pneumococcal infection^29^. In our study, G-CGSR antagonism reduced neutrophil trafficking into the BAL compartment by 30%, and this attenuated neutrophil response was sufficient to facilitate pneumococcal lung clearance. These findings also demonstrate a redundancy in the cytokine/chemokine network that coordinates neutrophil recruitment during pneumococcal infection, where G-CSFR contributes to trafficking.

The role of neutrophils during influenza viral infection has been reported in several studies, which highlighted their important protective role^9,30,31^. Whilst neutrophils confer substantial protection against viral propagation and dissemination^9^, this effect does not seem to be mediated by neutrophils *per se*. Rather, neutrophils appear to be crucial in modulating the expansion of anti-viral cytotoxic CD8+ T cells^30^. Additionally, it was recently revealed that neutrophils can release the T cell chemokine CXCL12 while migrating to the site of infection. This trail of CXCL12 provides a chemotactic gradient for subsequent cytotoxic CD8+ T cell migration^31^. Consistent with these reports^9,30,31^, we also found neutrophil depletion by 1A8 antibody was lethal in influenza A viral infection, accompanied with higher viral lung load (data not shown). In contrast, selectively antagonising G-CSFR signalling did not impact animal health status or body weight at any point during influenza A virus infection. More importantly, anti-G-CSFR therapy robustly dampened inflammation at the acute phase of infection (day 3) and promoted more rapid viral clearance at the later day 7 recovery phase.

In summary, our study confirms that the broad depletion of circulating and tissue neutrophils markedly increased the severity of lung pneumococcal infection. Whilst 1A8-mediated neutrophil depletion suppressed pathogen clearance, selectively antagonising G-CSFR was safe and effective at reducing neutrophilic inflammation and oedema without compromising lung clearance of the respiratory pathogens *S. pneumoniae* and influenza A virus in mice. The primary purpose of our study was not to develop a new therapy for acute respiratory infections, but rather investigate whether it is feasible to target G-CSFR without increasing risk of infection. A therapeutic monoclonal antibody against the human G-CSFR has recently completed a phase 1 clinical study (ACTRN #12616000846426) and our pre-clinical findings demonstrate feasibility of safely targeting this molecule as a strategy to reduce persistent neutrophilic inflammation in the lung. Hence, G-CSFR mAb therapy has the potential to reduce pathogenic inflammation and injury in chronic conditions such as asthma and arthritis without increasing susceptibility to common respiratory pathogens.

## Methods

### Animals

All experiments were approved by the RMIT University Animal Ethics Committee (AEC #1805) and performed in compliance with the National Health and Medical Research Council (NHMRC) of Australia guidelines. Eight weeks old female Balb/c mice were purchased from the Animal Resources Centre (Perth, Australia). In the first set of *S. pneumoniae* infection experiments, mice were prophylactically administered anti-G-CSFR mAb (100 µg, 5E2-VR81 clone, CSL Limited, Australia), anti-Ly6G mAb (100 µg, 1A8 clone, Bio X Cell, USA) or isotype control mAb (100 µg, CSL Limited, Australia) by intraperitoneal (i.p.) injection 24 hours (day -1) prior to intranasal inoculation with *S. pneumoniae* (EF3030, 19 F serotype, 1 × 10^5.0^ CFU – 3 × 10^6^ CFU in 35 µL saline). Mice were either culled 24 hours post inoculation (day 1) or treated with mAb on day 1 and day 3 and culled on day 2 and day 4 respectively. In the therapeutic experiments, mice were inoculated with *S. pneumoniae* (3 × 10^6^ CFU in 35 µL saline) and treated with mAb (100 µg i.p.) on day 2 post infection and outcomes were assessed on day 4 post infection. In the influenza A virus (IAV) infection experiment, anti-G-CSFR antibody or isotype control antibody treatments were performed as described above on day -1 prior to intranasal inoculation with IAV (HKx31, 100 PFU in 35 µL saline). Mice were either further treated with additional doses of the antibodies on day 1 and culled day 3 post infection or treated with 3 more doses of antibodies on day 1, day 3, day 5 and culled on day 7. Mice were humanely culled by pentobarbital overdosing, followed by tracheotomy and lavage. Bronchoalveolar lavage (BAL) and nasal lavage were performed using 1.3 mL or 0.4 mL PBS respectively. Flushed fluids were collected. Total BAL cells were enumerated, and differentials were determined using a Shandon™ Kwik-Diff ™ kit (Life Technologies, USA). Whole blood was withdrawn via cardiac puncture and analysed on a CELL-DYN Emerald haematology analyser (Abbott Core Laboratory, USA). Lungs were perfused with ice-cold PBS to remove residual blood. The left lobe of the lungs was excised and fixed in 10% neutral-buffered formalin for histology and the rest was snap-frozen in liquid nitrogen prior to −80 °C storage for further analysis.

### Quantification of *S. pneumoniae* and influenza A virus

Quantification of *S. pneumoniae* (CFU) in the BAL fluid (BALF), nasal wash and whole blood was performed by serial dilutions cultured overnight on selective agar (horse blood agar supplemented with 5 µg/mL gentamicin) at 37 °C with 5% CO_2_. Quantitative real time PCR (qPCR) on polymerase A subunit (PA) gene was performed on total RNA extractions to measure influenza A virus in the lung tissue using TaqMan® Fast Virus 1-Step Master Mix (Life Technologies, USA) as previously described^32^. Lung viral load is expressed as fold of change vs saline controls.

### Reverse transcriptase quantitative PCR (RT-qPCR) for gene expression analysis

Total RNA was purified from lung tissue using RNeasy kit (Qiagen, Germany), from which cDNA was prepared using High Capacity cDNA Kit (Life Technologies, USA). qPCR was performed using bioinformatically validated TaqMan probes (Life Technologies, USA). The threshold cycle values (Ct) were normalized to a reference gene (glyceraldehyde phosphate dehydrogenase; GAPDH) and the relative fold change determined by the ΔΔCt value.

### Myeloperoxidase activity and quantification of total protein levels in BALF

Myeloperoxidase activity was measured as a surrogate marker for tissue neutrophils as previously described^32^. BALF dsDNA (marker for netosis) was measured using the Quant-iT PicoGreen dsDNA Assay Kit (Life Technologies, USA) and net gelatinase activity was measured using the EnzChek Gelatinase/Collagenase Assay Kit (Life Technologies, USA) as previously described^32^. Total protein levels in the BALF were determined by a Pierce BCA Protein Assay Kit (Life Technologies, USA).

### Data analysis

Data are presented as the mean ± SEM except for bacterial/viral load which is presented as median ± interquartile range. All data were statistically analysed using GraphPad Prism 7.0 (Graphpad, San Diego, CA). Where detailed and appropriate, two-tailed Students t-tests or one-way analyses of variance (ANOVA) with Bonferroni’s post-hoc tests were used. p < 0.05 was considered to be statistically significant.
